# Vaccination coverage of triple viral and poliomyelitis in Brazil, 2011-2021: temporal trend and spatial dependency

**DOI:** 10.1590/1980-549720230047

**Published:** 2023-10-20

**Authors:** Isadora Gabriella Silva Palmieri, Lucas Vinícius de Lima, Gabriel Pavinati, José Arthur Paschoalotto Silva, Sonia Silva Marcon, Ana Paula Sayuri Sato, Gabriela Tavares Magnabosco

**Affiliations:** I Universidade Estadual de Maringá, Graduate Program in Nursing – Maringá (PR), Brazil.; II Faculdade das Indústrias, Residency in Artificial Intelligence – Londrina (PR), Brazil.; III Universidade de São Paulo, School of Public Health, Department of Epidemiology – São Paulo (SP), Brazil.

**Keywords:** Vaccines, Vaccine-preventable diseases, Anti-vaccination movement, Public health surveillance, Vacinas, Doenças preveníveis por vacina, Movimento contra vacinação, Vigilância em saúde pública

## Abstract

**Objective:**

To analyze the coverage of MMR and polio vaccines, the temporal trend and spatial dependence, in children up to one year of age in Brazil, between 2011 and 2021.

**Methods:**

Ecological study with secondary data on vaccination coverage rates, made available by the National Immunization Program Information System. Trend analysis was carried out using the joinpoint method, according to geographic regions, estimating the annual percentage change (APC) and its respective confidence interval (95%CI). Choropleth maps of distribution by health region were constructed and, subsequently, the spatial dependence was verified using Moran's statistics.

**Results:**

Between 2011 and 2021, vaccination coverage declined in Brazil, both for MMR (APC: −6.4%; 95%CI −9.0; −3.8) and for poliomyelitis (APC: −4. 5%; 95%CI −5.5; −3.6). There was a decline in coverage of both vaccines in all geographic regions over the years of the study, except in the South and Midwest for the MMR vaccine. Since 2015, few health regions in the country have achieved adequate vaccination coverage (≥95.0% to <120.0%). The North and Northeast health regions showed low-low clusters in the univariate analysis for both immunobiological.

**Conclusions:**

It is urgent to consider studies like this one for the planning of more effective strategies for immunizing children, especially in areas with higher falls. In this way, barriers to access to immunization can be broken, given Brazil's heterogeneity, and access to reliable information that increases confidence in vaccine efficacy can be expanded.

## RESUMO

**Objetivo:**

Analisar a cobertura das vacinas tríplice viral e contra poliomielite, a tendência temporal e a dependência espacial em crianças de até um ano no Brasil, entre 2011 e 2021.

**Métodos:**

Estudo ecológico com dados secundários das taxas de cobertura vacinal (CV), disponibilizadas pelo Sistema de Informação do Programa Nacional de Imunização. A análise de tendência ocorreu pelo método *joinpoint*, segundo regiões geográficas, estimando a variação percentual anual (APC) e seu respectivo intervalo de confiança (IC95%). Foram construídos mapas coropléticos de distribuição por região de saúde e, posteriormente, verificou-se a dependência espacial pela estatística de Moran.

**Resultados:**

Entre 2011 e 2021, as coberturas vacinais apresentaram queda no Brasil, tanto para tríplice viral (APC: −6,4%; IC95%: −9,0; −3,8) quanto para poliomielite (APC: −4,5%; IC95% −5,5; −3,6). Houve declínio da cobertura de ambas as vacinas em todas as regiões geográficas ao longo dos anos de estudo, exceto no Sul e no Centro-Oeste para a vacina tríplice viral. Desde 2015, poucas regionais de saúde do país atingiram a CV adequada (≥95 a <120%). As regiões sanitárias do Norte e do Nordeste apresentaram *clusters* do tipo baixo-baixo na análise univariada para ambos os imunobiológicos.

**Conclusão:**

É premente considerar estudos como este para o planejamento de estratégias mais eficazes à imunização de crianças, sobretudo em áreas de maior queda. Desse modo, pode-se romper as barreiras do acesso à imunização, dada a heterogeneidade brasileira, e ampliar o acesso a informações fidedignas que aumentem a confiança na eficácia vacinal.

## INTRODUCTION

In Brazil, the National Immunization Program (*Programa Nacional de Imunizações* – PNI), created in 1973 and coordinated by the Ministry of Health, has been consolidating itself as one of the most relevant interventions in public health worldwide. This is because, since its structuring, the PNI has had an impact on the morbidity and mortality profile of the population and has been molding itself to the changes that have occurred in the epidemiological, political, and social scenario^1^.

Despite this, the drop in vaccination coverage (VC) for children emerged as an issue of interest to public health since it is a child's right to health and, therefore, a collective duty^2^. It is known that vaccination eliminates or drastically reduces the risk of illness or serious manifestations of communicable diseases, which can lead to hospitalization or even death, avoiding structural and economic impacts on the health care system^3^.

In Brazil, 43 immunobiologicals are offered by PNI within the scope of the Brazilian Unified Health System (*Sistema Único de Saúde* – SUS), among which are the 19 vaccines of the basic childhood immunization schedule^4^. In this context, vaccines against polio, and against measles, mumps and rubella (triple viral – MMR) have been essential on the national scene, especially as they act in the prevention and control of highly contagious diseases^5^.

It should be noted that, thanks to the performance of the PNI, the country achieved the eradication of smallpox in 1973, wild poliomyelitis in 1989, congenital rubella in 2015, and neonatal tetanus in 2020^6^. In addition, the program provided control of several other diseases, such as measles, which, although it was eliminated in 2000, is a challenge today^5–8^.

Despite the relevant history that vaccination has, in terms of effectiveness in preventing diseases and injuries, vaccine acceptance is still not universal, and is often questioned and criticized^7,8^. It is known that sociodemographic factors, family dynamics, government and health policies, access to information and feelings of insecurity regarding technology and unwanted vaccine effects can influence vaccine acceptance among parental figures^9,10^.

Vaccination hesitation is recognized as a behavior that includes vaccine delay and/or refusal, associated with a feeling of insecurity about this decision^11^. Associated to this behavior, low vaccination coverage and the spread of false messages on social media, measles, which had been eliminated from the Americas for the second time in 2016, reappeared in 2018 from imported cases, resulting from the intense migratory movement from Venezuela^6,12^.

As VC decreases and vaccine-preventable diseases increase in incidence or threaten to re-emerge, there is an urgent need to understand this phenomenon. In this sense, the potential of ecological studies that monitor trends over the years and the geographic distribution of VC is considered, contributing to the identification of areas of greater vulnerability, evaluation of policies and development of more effective strategies^13^.

Thus, one must recognize the need to understand the epidemiological scenarios in Brazil, which reach continental proportions and interfere directly and indirectly in the quality of health care offered to the population, with the aim of helping to direct sensitive and equitable strategies. That said, this study aimed to analyze the coverage of the triple viral and poliomyelitis vaccine, the temporal trend and spatial dependence in children up to one year old in Brazil, between 2011 and 2021.

## METHODS

Ecological study that had as units of interest the geographic regions, the Federative Units (FU) and the health care regions (HR) of Brazil. Data on the coverage of triple viral and poliomyelitis vaccines were used, referring to the application in children up to one year old. These VC data were recorded in the National Immunization Program Information System (*Sistema de Informação do Programa Nacional de Imunizações* – SI-PNI), accessed by the SUS Department of Informatics (*Departamento de Informática do SUS* – DATASUS) on October 10^th^, 2022.

The SI-PNI has been implemented in Brazil since 2010 and aims to monitor the vaccination status of the population. However, as a new technology, its adhesion and acceptance depend on a series of factors so that it can be effectively implemented. In this sense, although it is an essential tool for Brazilian public health, it should be considered that there are places where the implementation of the SI-PNI is difficult or insufficient, mainly due to the lack of organization of production processes and/or training of people^14^.

Brazil has five geographic regions (North, Northeast, South, Southeast, and Center-West) and 27 FU, which, in the field of health care, are organized into 438 health regions made up of a group of neighboring municipalities that share cultural, economic, social, infrastructure and transport aspects, contributing to the organization of services. Such factors help the planning and execution of actions, guaranteeing resolutive access, in a timely manner and with quality^15^.

This study considered the records whose vaccine application date had occurred in the period from January 2011 to December 2021. For this purpose, VC data calculated by the SI-PNI itself were used, according to the formula: number of doses applied according to the indication (1^st^, 2^nd^ or 3^rd^ dose), divided by the target population, and the result was multiplied by 100. For MMR and poliomyelitis, the application of the 1^st^ and 3^rd^ dose was considered, respectively on annual data on live births, obtained by the Information System on Live Births (*Sistema de Informações sobre Nascidos Vivos* – Sinasc)^16^.

For trend analysis, the Joinpoint Regression Program, version 4.9.1, developed by the National Cancer Institute (Bethesda, Maryland, USA) was used. This software allows the analysis of time series using the point-to-point regression method, in which the variation was estimated by Poisson regression and significance tests by the Monte Carlo permutation method^17^. Calendar years were considered as the independent variable, and logarithmized VC rates as the dependent variable.

The regression model by inflection points (joinpoint regression) seeks to verify changes in the evolutionary trend and explain the series better than a straight line. These changes, the joinpoints, can be increasing or decreasing. To describe the change in trend, the annual percent change (APC) and its respective 95% confidence interval (95%CI) were calculated; trends were significant when the value did not cross the null point of the 95%CI (p≤0.05)^17^.

For spatial analysis, maps of the distribution of VC rates of immunobiologicals were constructed. For this purpose, the shapefile available on the Brazilian Open Data Portal was used. VC was grouped by the arithmetic mean into two four-year periods (2011–2014 and 2015–2018) and one three-year period (2019–2021), classifying them into:

1. Insufficient: <95%;
2. Adequate: ≥95 to <120%; and
3. Overrated: ≥120%^17^

This interval considered the recommended value to effectively contribute to the control, elimination or eradication of these diseases^13^.

Subsequently, the analysis of spatial dependence was carried out using univariate Moran statistics, which are subdivided into global and local Moran index. The global Moran index verifies the presence of spatial dependence of VC rates on a single parameter for the country. This value ranges from −1.00 to +1.00; values close to 1.00 indicate spatial, inverse (−) or direct (+) dependence, and when close to 0.00, data randomness. The pseudosignificance test was applied with 999 permutations^18^.

When the presence of significance was verified, the local Moran index was applied to portray the clusters: high-high (HH), areas and neighbors with high VC; low-low (LL), areas and neighbors with low VC; high-low (HL), areas with highs and neighbors with low VC; low-high (LH), areas with low and neighbors with high VC; and non-significant (NS), areas and neighbors without clear spatial expression^13^. Spatial analyses were performed using GeoDa^®^ software, version 1.20, and maps were constructed using QGIS^®^, version 2.36. A statistical significance level of 5% (p≤0.05) was adopted.

This study, in line with the ethical guidelines recommended by Resolutions No. 466, of 2012, and No. 510, of 2016, of the National Health Council, linked to the Ministry of Health of Brazil, waived consideration by the Ethics Committee in Research involving Human Beings, because it is a research carried out only with secondary data, which are available in public and unrestricted access, without identifying the participants.

## RESULTS

In the period from 2011 to 2021, the coverage of the studied vaccines varied widely in Brazil. The year with the highest VC was 2014 (112.8%) for the MMR vaccine (Table 1). The lowest VC in the country were in the year 2021, in the states of Rio de Janeiro (59.4%), Acre (60.2%), and Pará (62.7%). It is noteworthy that in these FU, in 2011, VC was around 100%. In 2021, for MMR, the best VC were in the states of Santa Catarina (86.5%) and Paraná (87.5%), so that the South Region had the best VC (Table 1).

**Table 1:** Vaccination coverage rates for the 1^st^ dose of the triple viral vaccine, according to the Federative Units of Brazil, between 2011 and 2021.

Location		2011	2012	2013	2014	2015	2016	2017	2018	2019	2020	2021
North	RO	102.7	105.4	106.5	146.9	109.0	109.8	103.0	101.7	106.4	84.2	82.5
	AC	105.2	90.2	95.0	99.2	84.2	75.7	75.1	83.1	87.4	60.2	60.2
	AM	94.6	103.3	98.7	114.4	95.4	83.6	79.8	89.8	92.1	77.0	73.1
	RR	97.9	87.8	89.0	110.2	108.5	90.8	86.5	99.3	81.2	69.5	67.3
	PA	109.2	102.2	98.4	115.7	71.9	69.6	67.5	77.3	82.8	62.4	62.7
	AP	93.0	91.5	95.6	113.2	89.0	97.4	72.0	77.1	87.2	52.5	64.2
	TO	91.4	91.6	102.2	105.5	94.7	91.9	83.3	91.2	91.1	82.5	80.5
Northeast	MA	112.8	98.2	106.4	123.9	90.5	80.0	76.9	84.0	87.1	64.3	63.3
	PI	97.0	98.3	102.4	93.1	81.2	81.5	77.8	87.8	89.0	78.5	75.0
	CE	110.9	97.6	115.2	140.7	110.8	119.8	100.7	111.5	103.0	91.8	74.5
	RN	99.4	98.6	113.1	110.3	95.0	96.1	75.6	88.7	93.7	79.0	72.6
	PB	102.3	92.7	114.6	120.4	93.7	96.6	90.9	96.7	105.7	80.4	70.9
	PE	113.6	104.5	120.8	108.4	97.8	112.7	96.4	104.7	101.0	79.2	72.0
	AL	89.9	93.1	110.7	113.2	98.7	102.2	99.2	107.2	106.2	82.6	77.7
	SE	98.0	97.3	111.4	94.6	92.0	92.1	83.2	95.5	91.0	76.2	76.5
	BA	100.2	97.9	109.2	114.9	90.2	85.7	79.2	82.3	84.7	79.7	66.3
Southeast	MG	100.8	104.9	108.3	109.2	100.1	98.9	89.3	97.5	97.0	93.1	82.3
	ES	103.7	105.1	106.2	109.4	99.0	104.3	83.8	95.5	95.0	87.8	80.8
	RJ	107.1	97.2	108.2	112.5	105.4	109.3	94.3	99.7	96.6	61.6	59.5
	SP	100.3	99.5	103.4	105.0	97.9	93.0	86.7	91.5	91.8	86.8	77.9
South	PR	98.5	99.9	110.2	113.4	99.4	91.9	88.1	89.8	92.0	86.2	86.5
	SC	99.7	100.4	104.6	112.2	103.4	99.0	91.8	92.1	95.8	87.6	87.5
	RS	93.8	91.6	105.7	107.7	87.8	90.5	83.3	88.7	91.2	86.1	79.9
Center-West	MS	96.2	100.8	113.9	143.8	112.5	101.0	91.2	104.5	104.9	81.1	78.1
	MT	98.4	99.3	107.8	120.7	98.7	96.7	85.3	89.8	89.9	82.9	80.9
	GO	115.5	107.7	117.8	122.1	94.8	85.9	81.0	87.8	88.4	76.6	79.5
	DF	89.5	92.9	105.2	104.9	67.6	131.8	78.5	86.3	85.7	82.2	83.2

Source: National Immunization Program Information System.

With regard to poliomyelitis, the highest VC in the country was in 2011 (101.3%). Subsequently, there was a decline, reaching 42.6 and 45.3% in 2020 and 2021, respectively. The state of Amapá registered the lowest VC in the country in the period, followed by Rio de Janeiro (56.8% in 2020 and 55.7% in 2021). When compared to the other regions, once more, the North had the lowest VC, with the exception of the state of Tocantins (80.2%). The South Region had the best VC in 2021: Paraná (80.7%) and Santa Catarina (83.7%), with values above those of the rest of the country (Table 2).

**Table 2:** Vaccination coverage rates for the 3^rd^ dose of polio vaccine, according to Brazilian Federative Units, between 2011 and 2021.

Location		2011	2012	2013	2014	2015	2016	2017	2018	2019	2020	2021
North	RO	107.3	105.7	100.1	108.1	105.4	105.4	108.2	101.9	98.3	82.5	74.6
	AC	111.0	96.1	92.8	75.4	82.7	71.3	74.0	78.3	81.7	63.1	61.8
	AM	87.2	91.9	96.8	98.9	104.8	76.2	76.4	79.3	83.3	68.2	67.8
	RR	95.5	88.8	86.5	89.5	112.3	88.5	90.5	79.8	79.8	73.7	51.0
	PA	101.5	97.8	97.0	84.4	72.1	63.2	67.6	69.1	72.7	59.8	56.8
	AP	84.6	92.7	93.3	80.7	92.4	47.6	63.2	68.7	73.0	42.7	45.4
	TO	99.6	92.8	97.0	90.5	97.2	84.8	86.1	91.7	88.2	84.4	80.2
Northeast	MA	102.9	97.5	105.7	93.4	100.0	69.4	74.3	80.6	75.7	60.9	62.0
	PI	98.5	93.9	93.1	81.9	80.9	70.7	78.1	83.7	81.9	73.0	72.8
	CE	100.0	97.8	104.8	103.9	111.7	107.9	97.3	111.1	93.5	88.4	74.3
	RN	96.9	94.0	93.9	95.6	97.6	70.3	69.5	90.3	80.7	70.6	71.7
	PB	102.0	92.1	108.2	100.4	96.1	85.5	82.3	92.3	92.6	73.8	70.3
	PE	109.6	100.1	101.1	101.7	109.3	90.4	84.7	94.7	85.6	72.8	69.0
	AL	90.6	90.2	97.5	93.3	94.3	80.1	83.9	96.1	87.9	74.4	77.5
	SE	103.4	96.9	99.4	94.3	93.7	78.3	79.0	89.7	80.9	71.6	72.3
	BA	97.3	93.3	96.4	93.9	95.4	70.7	78.3	78.3	74.8	70.9	63.1
Southeast	MG	103.2	98.6	103.2	96.5	97.1	88.3	87.1	97.8	88.5	86.6	76.5
	ES	108.3	104.9	100.2	101.3	99.4	89.3	83.2	91.0	86.7	81.7	77.4
	RJ	112.2	96.9	100.0	100.9	107.0	89.9	88.8	87.5	73.6	56.8	55.8
	SP	100.3	96.4	99.0	95.7	99.7	83.8	87.7	92.6	86.6	82.3	74.4
South	PR	102.5	96.8	104.7	98.8	97.4	87.5	90.4	90.9	89.7	86.4	80.8
	SC	101.2	100.0	97.8	97.2	102.4	92.1	95.1	94.6	93.7	88.7	83.8
	RS	95.1	89.4	100.3	95.4	89.2	84.5	85.7	85.7	85.1	85.1	76.4
Center-West	MS	95.5	102.3	118.0	130.1	120.4	93.8	91.5	96.0	94.4	83.2	75.7
	MT	103.2	99.5	101.2	102.5	102.8	90.6	84.1	90.3	85.8	81.5	76.3
	GO	107.1	101.0	107.7	97.7	95.9	82.1	81.5	85.5	81.5	78.1	72.7
	DF	86.5	93.7	112.2	94.3	74.9	136.8	84.4	86.0	84.3	81.5	73.2

Source: National Immunization Program Information System.

The decreasing trend of VC for MMR in Brazil was (APC: −6.4; 95%CI −9.0; −3.8), being more pronounced in the Northeast (APC: −8.2; 95%CI −10.8; −5.6). The South and Center-West regions showed a stationary trend. For poliomyelitis, the country also showed a downward trend (APC: −4.5; 95%CI −5.5; −3.6) and all geographic regions had a decrease, with emphasis on the North Region, which had the highest negative variation (APC: −6.3; 95%CI −6.3; −4.6) (Table 3).

**Table 3:** Temporal trend of vaccination coverage for the 1^st^ dose of the MMR vaccine and for the 3^rd^ dose of the polio vaccine, according to cutoff points obtained by the joinpoint regression method, according to Brazil Macroregions, between 2011 and 2021.

Location		Period	APC* (95%CI^b^)	p-value*	Trend
MMR	Brazil	2011–2021	−6.4 (−9.0; −3.8)	<0.001†	Decreasing
	North	2011–2021	−6.3 (−10.5; −1.9)	0.011†	Decreasing
	Northeast	2011–2021	−8.2 (−10.8; −5.6)	<0.001†	Decreasing
	Southeast	2011–2021	−6.8 (−9.2; −4.2)	<0.001†	Decreasing
	South	2011–2021	−4.4 (−10.4; 1.9)	0.116	Stationary
	Center-West	2011–2021	−6.9 (−15.0; 1.8)	0.118	Stationary
Poliomyelitis	Brazil	2011–2021	−4.5 (−5.5; −3.6)	<0.001†	Decreasing
	North	2011–2021	−6.3 (−6.7; −4.6)	<0.001†	Decreasing
	Northeast	2011–2021	−5.9 (−7.2; −4.6)	<0.001†	Decreasing
	Southeast	2011–2021	−4.1 (−5.0; −3.2)	<0.001†	Decreasing
	South	2011–2021	−2.2 (−3.2; −1.3)	<0.001†	Decreasing
	Center-West	2011–2021	−4.0 (−5.1; −2.8)	<0.001†	Decreasing

APC: annual percentage change; 95%CI: 95% confidence interval (minimum; maximum).

* value by Monte-Carlo permutation test;

† statistically significant value.

Source: National Immunization Program Information System.

**Figure 1:**
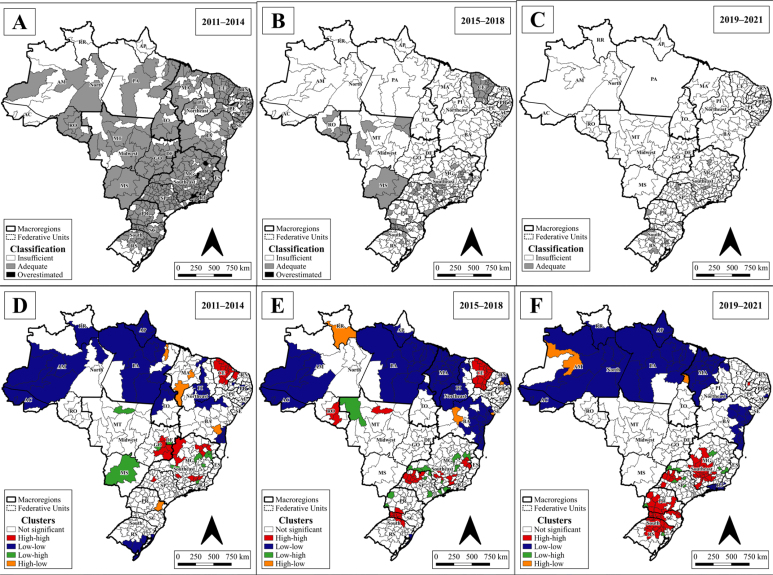
(A, B, and C) Spatial distribution of vaccination coverage classification as insufficient (<95%), adequate (≥95 to <120%), and overestimated (≥120%); and (D, E, and F) of clusters of the univariate local Moran index, on the 1^st^ dose of the MMR vaccine, according to health regions in Brazil, between 2011 and 2021.

Source: National Immunization Program Information System.

It could be observed that the HR of the states of Roraima, Amapá, and Acre had insufficient VC throughout the period. Other states, such as Amazonas, Pará, Mato Grosso, Maranhão, Piauí, Bahia, and Rio Grande do Sul, also had some regions with a VC below the recommended level. The health regions of Amazonas, Pará, Acre, and Amapá gathered most of the low-low clusters. High-high clusters were found in the states of Goiânia, Ceará and in the South Region (Figure 1).

In the second triennium (2015-2018), MMR VC dropped across the country. Almost all HR in the North and Northeast did not reach an adequate VC. Minas Gerais, Ceará, and Rondônia had some HR with adequate VC. In the last time frame (2019-2021), the whole country had a drop in VC, and most reached insufficient levels, except for some HR in Rio Grande do Sul, São Paulo, and Minas Gerais. Low-low clusters spread further in the North and Northeast (Figure 1).

**Figure 2:**
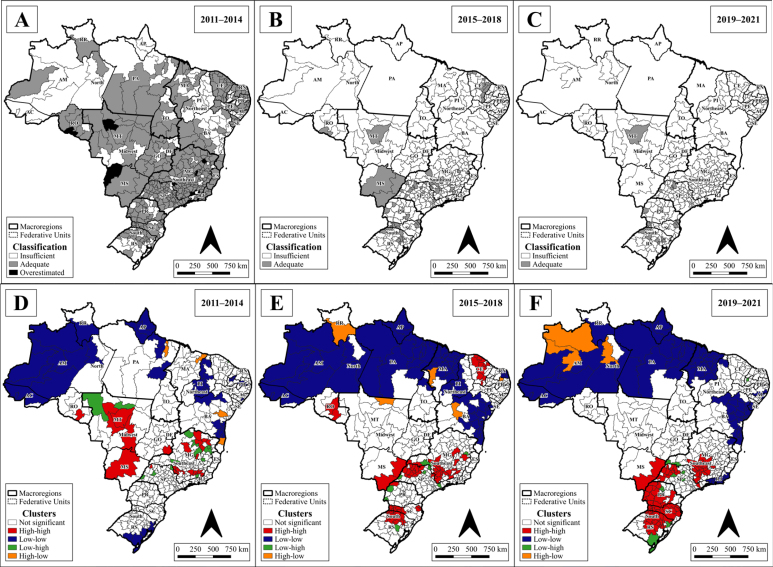
(A, B, and C) Spatial distribution of vaccination coverage classification as insufficient (<95%), adequate (≥95 to <120%) and overestimated (≥120%); and (D, E, and F) of clusters of the univariate local Moran index, on the 3^rd^ dose of polio vaccine, according to health regions in Brazil, between 2011 and 2021.

Source: National Immunization Program Information System.

As for the VC of the poliomyelitis vaccine, since the first triennium (2011–2014) unsatisfactory values were observed, which, in the spatial analysis, were more evident among the HR of the North, Northeast and extreme South regions. However, this time frame showed satisfactory values in most of the FU. In the univariate analysis by Moran, the North Region presented low-low clusters, as well as in some points in the Northeast and in the extreme South of the country (Figure 2).

The subsequent triennium (2015–2018) showed a decrease in the VC of the 3^rd^ dose of the polio vaccine. Finally, the 2019-2021 triennium showed low VC throughout the territory, with the exception of a few HR. Among the areas with adequate coverage, the border territory of the states of the Southern Region, the north coast of Santa Catarina, the northwest of the state of São Paulo and one HR of the state of Mato Grosso stood out. Low-low clusters prevailed in the North and Northeast, with some high-low transition regions (Figure 2).

## DISCUSSION

The findings of the present study demonstrate a decreasing trend of poliomyelitis and MMR VC across the country, from 2011 to 2021. Other studies have endorsed the concern with the drop in VC rates in Brazil, a movement that affects several immunobiological agents^19–21^. Therefore, the importance of studies capable of identifying the regions most affected by the decrease in childhood immunization is reinforced, in order to plan effective strategies with a view to reversing the current scenario.

The North Region was the most strongly affected by the decrease in the VC of the analyzed immunobiologicals. Among the possible causes, the association between economic status and adherence to childhood vaccination is considered. In this logic, it should be noted that the North Region has the lowest Gross Domestic Product (GDP) in the country, an indicator that summarizes economy, helping to understand performance^22^. This demonstrates the predominance of low VC in FU with worse socioeconomic indicators^23^ and the low response capacity of these locations in the context of economic and social crisis, such as the one in force in the country since 2015.

Thus, it is recognized that the higher the socioeconomic level of the municipality or region, the degree of urbanization and the GDP, the higher the probability of achieving the recommended VC, disseminating health information, publicizing vaccination campaigns and offering quality services^24^. This may be associated with different municipal, regional, and state public policies, which end up inferring the availability of health resources for the population, including vaccination^25^.

A multicenter study carried out in municipalities of the five regions of Brazil, between 2013 and 2014, in primary care services, identified that the North Region has the highest proportion of units that do not have a specific room for vaccination, presenting precarious conditions and variation in the availability of vaccines, which reveals social and structural differences, a result of public investments in health, and which contributes to the occurrence of vaccine-preventable infectious disease outbreaks^26^.

In view of this, it is worth recalling the records of measles cases in 2020 in the states of Pará and Amapá, with approximately 5,492 confirmed cases and eight deaths, and also in 2021, when more than 96% of cases were concentrated in these FU^6^. Here, it should be noted the intense migratory movement on the border between the North Region and Venezuela, which, in addition to favoring the transmission of communicable diseases, interferes with access to care and health information for the contingent of immigrants^7^.

Furthermore, it is known that there are regional inequalities in socioeconomic and programmatic development in Brazil^25^. Among the aspects that constitute barriers to access to health, one can mention geographic inaccessibility, which refers to distance, travel time and the means of transport necessary for the health service; the availability of services in appropriate quantity and quality; and the acceptability and relationship between professionals and users^27^.

Distance and irregularities on commuting to health services, including living in rural areas, are recurrent reasons for low demand for vaccination^28^. It is noteworthy that 25% of residents in rural areas in Brazil are in the Northeast^28^ and that the territorial extension of the North causes long periods of displacement and transport costs^27,28^. The South and Southeast have fewer of these barriers^27^. These disparities can influence the VC between the regions of the country and, therefore, urgently need to be considered in the planning of management and health care by municipal, regional, and state managements.

Still, the fragility of the family and social network can constitute a barrier to access to childhood vaccination^29^. As evidenced in a study by the World Health Organization (WHO), social components, family support, access to information and vaccine recommendation by health professionals can impact the population's perception of risk, as well as tend to provide knowledge and understanding of the benefits of immunization, building confidence in the effectiveness of vaccines^10^.

In Brazil, it is necessary to consider the role of some government policies in maintaining and improving VC rates. The *Bolsa Família* Program (*Programa Bolsa Família* – PBF), which provides for the transfer of income to people in poverty and extreme poverty, demands, among the eligibility criteria, compliance with the basic childhood vaccination schedule^30^. As a result, PBF can help improve adherence to childhood vaccination, especially among people of lower socioeconomic status who are covered by the policy^31^.

It should also be mentioned that since 2012, multivaccination campaigns have been carried out annually, organized by the Ministry of Health and by the State and Municipal Health Departments, which generally take place outside business hours. This action seeks to expand the population's access to vaccines and update the vaccination status of children and adolescents^32,33^. However, it is emphasized that this strategy does not exempt parents from responsibility^34^ and depends on understanding and political interest in providing these actions and safe information^35^.

Despite the effectiveness of these strategies, it was evident that the drop in VC reached all regions of Brazil, starting, mainly, in 2015. This period was crossed by changes in the country's economic policy, which affected public health. Such a drop was accentuated in the period from 2018 to 2021, concomitantly with a government policy that disqualified vaccination and cut spending on financing the sector. As an example, there are the restrictive measures imposed on the SUS, such as Constitutional Amendment No. 95, which proposed the freezing of public spending for 20 years^36^.

Also during this period, in addition to discouraging vaccination and the dissemination of false news by the Federal Government for the 2018-2022 quadrennium, isolation due to Covid-19 restricted or interrupted the activities of primary care units, while others became place of testing and treatment for the disease. This context of service overload may have made it difficult or impossible to develop other actions, such as vaccination^34,35,37^.

Furthermore, in addition to negatively influencing routine vaccination strategies, the pandemic has caused people to fear contracting the virus. This protective instinct may have caused a decrease in demand for non-urgent health services^34,35,37^. However, a study carried out in African countries identified that routine vaccination and the possible exposure of children before the breakdown of isolation represented greater benefits than harm, when compared with non-vaccination^37^.

Although it is recognized that the use of disservice government policies in the face of programs and strategies already consolidated in recent years, combined with the dissemination of untrue news in information media and social media and the Covid-19 pandemic, may have influenced the drop in VC, it should be noted that the false sense of security due to the apparent decrease in the number of people with vaccine-preventable diseases and/or their sequelae can also lead to a decline in vaccination. In this context, it is understood that vaccine hesitancy is linked to confidence in immunobiologicals and the complacency of the population regarding the low perception of the risk of illness^38^. This whole scenario could justify the alarming findings of this research and indicate paths for the resumption, reinsertion, and insertion of public policies, programs and actions that seek, in fact, to protect individual and collective health, and to safeguard the state of social well-being of Brazilians.

Finally, it should be noted that, in addition to presenting adequate VC rates, it must be homogeneous to achieve immunization efficiency^20^. In this context, this research, by identifying the highest priority regions, can collaborate with the elaboration of public policies aimed at regional needs and specificities, as well as improving immunization strategies, according to the respective epidemiological scenarios of VC. In addition, it reinforces the need for political commitment in training health and education professionals among the population, with a view to recovering and disseminating reliable information about immunobiologicals to curb the advancement of the reemergence of diseases, such as those mentioned above. Furthermore, it is up to governments to launch efforts to regain social policies and health promotion programs with the intention of rebuilding conceivable standards of health and well-being of the population.

It is pointed out, however, that this research must be interpreted with some limitations. The use of secondary data represents one of these weaknesses, as they are subject to incompleteness and filling errors. In addition, there is the possibility of underreporting applied doses and underestimation of the denominator, which may compromise the calculation of the VC, especially in the context of the Covid-19 pandemic. Thus, it is understood that the data presented may not reflect the real epidemiological scenario, raising the need for further studies.

In short, there was a decreasing trend in childhood VC for poliomyelitis and MMR in Brazil between 2011 and 2021. Heterogeneity was identified in vaccination between Brazilian regions, so that the North and Northeast regions were the most affected by the drop in VC. Still, it was seen that until 2015, VC was mostly adequate; however, throughout the series, few regions had acceptable vaccination rates. These findings signal an alert to the risk of spreading these diseases and require immediate intervention by health authorities.
